# The Effects of Smartphone Spectatorship on Attention, Arousal, Engagement, and Comprehension

**DOI:** 10.1177/2041669521993140

**Published:** 2021-02-20

**Authors:** Kata Szita, Brendan Rooney

**Affiliations:** Aalto Behavioral Laboratory, 174277 Aalto University, Espoo, Finland; Department of Cultural Sciences, 3570 University of Gothenburg, Gothenburg, Sweden; School of Psychology, 8797 University College Dublin, Dublin, Ireland

**Keywords:** smartphones, screen size, movie, attention, presence, comprehension, eye movements, electrodermal activity

## Abstract

The popularity of watching movies and videos on handheld devices is rising, yet little attention has been paid to its impact on viewer behaviour. Smartphone spectatorship is characterized by the small handheld screen as well as the viewing environment where various unrelated stimuli can occur, providing possible distractions from viewing. Previous research suggests that screen size, handheld control, and external stimuli can affect viewing experience; however, no prior studies have combined these factors or applied them for the specific case of smartphones. In the present study, we compared smartphone and large-screen viewing of feature films in the presence and absence of external distractors. Using a combination of eye tracking, electrodermal activity measures, self-reports, and recollection accuracy tests, we measured smartphone-accustomed viewers’ attention, arousal, engagement, and comprehension. The results revealed the impact of viewing conditions on eye movements, gaze dispersion, electrodermal activity, self-reports of engagement, as well as comprehension. These findings show that smartphone viewing is more effective when there are no distractions, and smartphone viewers are more likely to be affected by external stimuli. In addition, watching large stationary screens in designated viewing environments increases engagement with a movie.

## Introduction

The peculiarity of smartphone spectatorship lies in its pervasiveness, the fact that it has little in the way of cultural, behavioural, temporal, or spatial constraints. Viewing experiences on smartphones differ from cinematic and home video experiences in a number of features. These differences can be classified in two main ways. First are the device-related differences; the screen is smaller, and the viewer has a bodily connection to the device. That is, they hold the device in their hands and may adjust its position and operations through haptic interaction. Second, the viewing environment (the location context in which the viewing occurs) is predominantly an unenclosed space where various unrelated activities and stimuli can occur in parallel to spectatorship, providing possible distractions from viewing.

One might argue that smartphones’ design and the viewing environments lead to less focused and less engaged viewing, where the smartphone viewer’s attention is divided between the movie and the physical space. If so, these features will have implications for gaze behaviour and narrative experience. Previous research has not explored the combination of these effects. Therefore, the aim of this paper is to explore how smartphone spectatorship would impact attention, arousal, engagement, and comprehension in comparison with large-, stationary-screen viewing. Over the next paragraphs, we review the ways in which the device’s features and external distractions may affect viewing experiences.

## 

### Screen Size and Handheld Control

Screen size and handheld control are perhaps the most vital features that define smartphone spectatorship and smartphone use in general. Screen size affects the proportion of the viewer’s visual angle that the screen and screened content cover, which has implications on attention to the screen, arousal, the sense of narrative presence, and comprehension. Previous studies that compare observing audiovisual stimuli on screens of different sizes demonstrate that larger screens produce higher levels of self-reported presence (IJsselsteijn et al., 2001; Lombard, Ditton et al., 1997; Slater & Wilbur, 1997; Troscianko et al., 2012) and emotional arousal (Lombard, Reich et al., 2000), improve completing visual tasks (Tan, 2004), enhance the sensation of reality (Hatada et al., 1980), and increase gaze dispersion on the screen (Smith, 2014). Similarly, greater immersion in video game play has also been reported when using larger screens (Bakdash et al., 2006; Hou et al., 2012).

In a study monitoring movie spectatorship, Troscianko et al. (2012) concluded that large screens produce higher subjective presence scores, especially during scenes depicting faces, even when the visual angles are constant, that is, when “big” screen viewers watch the screen from a greater distance than “small” screen viewers. Results of these studies imply that the attention paid to certain visual elements varies between screen size, which affects users’ or viewers’ emotional engagement with and sense of presence in a moving-image stimulus. Although these studies show that bigger is generally better in creating immersive and emotionally loaded experiences, there is insufficient evidence, not only on screens smaller than approximately 10 inches but even on the effects of manual control and movie or video viewing in environments where distractions are present.

Serving as a notable exception from the lack of research into small handheld screens, Bracken et al. (2010) found that the sense of spatial presence in a fictional environment is greater when watching a 32-inch television set compared with viewing a 2.5-inch iPod. Interestingly, their results also demonstrated that, although screen size showed no main effect on the other indices of presence, participants felt more immersed when watching a fast-paced action scene on the iPod than on the large screen.

Screen size raises the question of engagement and distraction even in that images (through, for instance, close-ups or wide-angle shots) change from being enlarged from their size in real life to being compressed, which may alter a movie’s affective qualities compared with large-screen viewing. Yet, the size of smartphones allows for handheld usage, which may increase the sense of engagement due to haptic control (IJsselsteijn et al., 2000) and to the possibility for adjusting the visual angle to an *ideal* degree (van Laer et al., 2014). This proprioceptive element projects a different rate of bodily involvement than in the case of fixed-screen viewing.

Haptic control in smartphone spectatorship implies a novel type of interactivity that, instead of being limited to predefined instances, is based on a viewer’s personal preferences and reactions to distractions in any moment (Szita, 2020). Smartphone users are able not only to adjust the screen position but even the image content or sound intensity on the smartphone interface. However, measuring the effects of such level of haptic interactivity presupposes methodological challenges due to the difficulties for reproducibility, which is perhaps the reason for the lack of research into this issue.

Studies on enjoyment, engagement, and empathy regarding interactive movies, movie trailers, and cross-media gaming (Ghellal & Lindt, 2008; Hu & Bartneck, 2008; Oh et al., 2014) touch upon questions of whether interactions would allow comprehending a coherent narrative and the way they affect engagement with a fictional world. In one of these studies, Oh et al. (2014) found that with the increase of viewer control, the sense of presence as well as mental and emotional involvement decreases depending on the type of content. In Vorderer et al.’s (2001) work, a similar tendency was observed, however, conditional to cognitive capacities. Although these results suggest a negative outcome of interactions during movie watching, they cannot fully predict the effects of smartphone viewing, where hapticity and freedom of control suggest increased engagement.

### Distractions

Cinema, television, and even computer screens are not only larger but also most commonly placed in a fixed position in spaces which are set to enhance viewing experience. Contrarily, smartphone users often consume moving-image content on their handheld devices as a secondary activity and in spaces not necessarily designed for movie watching. This increases the chance for environmental distractions that can be of various modalities, intensities, types, and can hold different amounts of relevance for a viewer.

In terms of spectatorship, few studies deal with the specific case of environmental distractions. Distraction conditions in previous research are often created by assigning secondary tasks, such as identifying difficult words (Tal-Or & Papirman, 2007) or paying attention to film language, such as editing (Tisinger, 2004) or the number of scenes in a movie sequence (for an overview, see Tukachinsky, 2014; Zhang et al., 2007).

Zwarun and Hall’s (2012) study serves as an exception in that it modelled online movie watching that may happen in unenclosed spaces or while engaging in other activities. The authors compared viewers’ engagement (narrative transportation) with a movie sequence when watching it in a low or high distraction environment. In the low distraction condition, participants watched the clip using noise-cancelling headphones, while in the high distraction condition, environmental noises and occasional onscreen messages distracted viewing. Zwarun and Hall’s findings indicate that high distraction viewing makes it more difficult to understand a movie and it decreases narrative transportation.

Introducing “deviant” and potentially distracting stimuli while completing visual tasks, other studies offer insights about attention and performance during distractions (Alho et al., 1997; Escera et al., 1998, 2000)—although not for the case of complex audiovisual stimuli, such as movies. Escera et al. (1998), for instance, observed that sound effects of varying relevance to a visual task lead to changes in reaction time and task performance. According to their results, newly introduced external sound increases reaction time and deviant sounds decrease performance. This effect can even be explained by that incongruent stimuli (such as Escera et al.’s deviant sound or unrelated distractions during movie watching) redirects attention from the main task or stimulus, but when the main task or stimulus demands high perceptual load, there are less available resources for directing attention to distractions (Lavie, 1995; Lavie et al., 2004). Works in video game studies about engagement with complex multimedia material and tasks amid environmental distractions support this idea, although centre on the elaborateness of audiovisual stimuli: For instance, in unison with the findings of Bailenson and Yee (2006), de Kort and IJsselsteijn (2008) explain that perception of the physical surroundings depends on how engaging are the mediated environment and the mechanisms of gameplay.

### Methodological Issues

According to the aforementioned findings, screen size, eventual interactions with a moving-image content, and distractions may have effects on attention patterns, emotional engagement, and performance (i.e., comprehension). Although the aforementioned studies touch upon some of the effects of screen size, handheld control, and visual and sonic distractions on attention and narrative experiences, there is a lack of research into the spectatorial behaviour of smartphone users. Another major shortcoming lies in the methodological solutions used.

For mapping attention to moving images, an applicable method is eye tracking (Duchowski, 2007; Mital et al., 2011). As feature films exogenously control attention to a significant extent through visual cues, such as lighting and motion (Itti, 2005; Mital et al., 2011), synchrony across viewers’ visual attention is generally expected to be high, which allows for testing the major effects of smartphone spectatorship on gaze behaviour (Smith, 2006; Smith et al., 2012). Eye tracking is also suitable for assessing differences in eye movements on screens of different sizes (Smith, 2014) and can inform conclusions regarding mental workload (May et al., 1990).

While eye tracking can provide sufficient information about visual attention (i.e., viewers’ gaze behaviour when watching a movie), this does not necessarily reflect top-down cognitive processes related to comprehending or engaging with an audiovisual narrative. As Loschky et al. (2015) and Hutson et al. (2017) demonstrate, gaze and information intake are generally highly correlated when watching movies; however, visual attention is less affected than narrative comprehension when manipulating the viewing context. Another limitation, specific to moving images, is the centre bias: Gaze tends to shift to the centre of the screen after cuts and when there is nothing attractive elsewhere (Tseng et al., 2009). Thus, gaze data do not tell us about comprehension.

Based on the limitations of eye-tracking measurements, additional methodological constructs can be used for assessing viewers’ emotional engagement with a movie and their comprehension of its narrative. Electrodermal activity (EDA) is a sensitive marker for arousal. Confrontation with emotionally loaded stimuli induces changes in, among other things, pulse and thermoregulation, and activates sweat glands. EDA signals these autonomic, unconscious, changes in skin conductance, providing information on emotional arousal, reactiveness, attention, and immersion (Boucsein, 2012) and can be used for monitoring reactions during movie watching (Potter & Bolls, 2012; Rooney et al., 2012).

EDA measures can capture event-related physiological reactions and are suitable for recording reactions during completing a task (e.g., watching a movie) without disruption. But while EDA can record momentary physiological changes, increase in sweat gland activity may be attributed to visual or sonic stimuli, narrative context, or even distractions. In other words, EDA responses derived from engaging with complex stimuli may be ambiguous.

Unlike EDA, self-report measures can tap into the subjective experience of engaging with the components of audiovisual media products. Widely used in research of media experiences, such as movies or virtual reality (Lombard, Ditton et al., 2000; Rooney & Hennessy, 2013; Troscianko et al., 2012), self-report measures can measure viewers’ overall experience and comprehension. Discrete (postexperiment) self-reports of engagement (e.g., sensation of presence, emotional engagement) and comprehension tests (multiple-choice or free-text tests) can provide feedback regarding the average value of engagement for a viewing session (subjective ratings of the overall experience) and comprehension/recollection of narrative details (presented once at given time segments).

It is, however, important to note that self-report measures, by their nature, are subjective, and respondents may be biased by social and cultural expectations or demand characteristics. Moreover, while data recorded using postexperiment self-reports can present the overall viewing experience and how much information a viewer absorbs, due to the interim between watching and responding, they may not capture objective information. Completing a complex task, for instance, watching a movie sequence requires meaning construction, containing both emotional and semantic components. Therefore, mental abilities, such as memory and language knowledge, can consequently influence self-reporting.

## The Present Study

Studies mentioned earlier show that a screen’s attributes and environmental stimuli can affect viewers’ gaze behaviour and narrative experience, which suggests that it is valuable to compare smartphone and regular screen viewing. Such a comparison is vital to distinguish the effects of smartphones and viewing environments during movie watching. This work can illuminate the convergence of media-consumption habits and provide knowledge of how modern digital media tools may impact moving-image experiences. In the lack of existing research to assess the specificities of smartphone spectatorship, we aim to fill a gap by measuring the impact of screen type and distractions on attention, arousal, engagement, and narrative comprehension using eye tracking, EDA measures, self-reports, and comprehension tests.

The present study recreated the common specificities of smartphone spectatorship and compared this to viewing a larger, stationary screen. Therefore, participants’ responses and experience were recorded while watching movie sequences on a smartphone or a fixed projector screen in the presence or absence of additional sonic and visual distractions.

Taking screen type and environmental distractions into account, we sought answers to the following research questions: How do screen type and distractors interact to affect the physiological measures of (a) attention and (b) arousal as well as (c) the self-reported indices of engagement and (d) narrative comprehension?

In accordance with previous research, we hypothesized that—as a result of the small size and handheld use of the screen—smartphone viewers in the presence of distractors would be less likely to maintain constant focus on the movie than large-screen viewers without distractions. Consequently, viewers’ gazes were expected to travel longer and leave the screen for a longer proportion of the trial time in the presence of distractions and when using the mobile screen. On one hand, this assumption was grounded in the idea that visual and sonic distractors would draw attention away from the screen. On the other hand, we predicted that the smaller the visual angle a screen covers leads to less focused and engaged viewing of a movie. This assumes that the mobile screen and the distracting stimuli induce more intense attention oscillation between the screen (the moving-image content) and the surrounding space and other stimulus sources.

Less focused attention on the screen and its content was hypothesized to negatively affect arousal and engagement with the movie (EDA and self-reported engagement). In addition, we even expected that participants in interrupted conditions and using the smartphone would score lower in the narrative comprehension test.

## Method

### Design

The experiment followed a two-by-two factorial design, where screen type (mobile screen and stationary projector screen) and the presence or absence of distractors were the two independent variables. This delivered four conditions: interrupted and uninterrupted mobile condition and interrupted and uninterrupted projector condition. To keep the conditions comparable, yet avoid sequential effects of viewing the same stimuli,^1^ we used two different, but similar sequences from the same movie (see later), where one sequence was always used for the interrupted conditions and the other for the uninterrupted conditions. Each participant watched both film clips, one on each type of screen, with and without distractions (interrupted mobile and uninterrupted projector or uninterrupted mobile and interrupted projector). This required an incomplete mixed design, in that participants were assigned to two of the four conditions. The order of the measurement conditions was randomized but counterbalanced to produce an equal number of trials for each combination. The incomplete design allowed for the research to retain the benefits of both between groups (avoiding sequential effects) and within subjects (participants acted as their own control). The limitations of the incomplete design were accounted for in the analysis.

### Film Stimuli

For the experiment, a contemporary Hollywood-style feature film, *The Walk* (Zemeckis et al., 2015), was used. *The Walk* is based on the true story of Philippe Petite, a French artist, who in 1974 performed a tightrope-walking act, completing several crossings illegally between the tops of World Trade Center’s towers. The storytelling style of classical and postclassical Hollywood films serves as a suitable starting point for investigating viewer behaviour and narrative information acquisition on smartphones. In addition to this, representing the most significant factors for exogenous control, the following criteria were taken in account when choosing the movie and selecting the relevant clips for the experiment. Besides the movie’s relative obscurity, yet up-to-date visual style (it needed to be recent and/or set in our present time or a relatively near past), another requirement was that it features details that maintain and control attention in an analogous way for all viewers. These details include short, fast-paced shots, semantically meaningful elements, such as facial expressions, landmarks, animate and moved objects, and congruent cultural references that induce identical and synchronous reactions (see Carmi & Itti, 2006; Cutting et al., 2011; Hasson et al., 2004; Itti, 2005; Mital et al., 2011; Smith & Henderson, 2008; Zacks & Magliano, 2011).

Two, each approximately 9 minutes, sequences were used for the experiment from the final section of the movie, where Petite, with the help of his “accomplices,” installs the wire and performs his walks. The two chosen clips were selected to fulfil the aforementioned criteria by including semantically meaningful elements, a variety of saliences and shot lengths, a wide range of emotions, and cross-media references (e.g., written texts) to the same extent. The selected parts of the movie were also required to evoke strong emotional reactions, without being violent or showing disturbing content. Despite the fact that they are mild enough not to cause discomfort, the two clips can evoke concerns for the protagonist or even moderate symptoms of acrophobia caused by the sight of the tall buildings or the deep void beneath the World Trade Center towers.

The two sequences depict two separate segments of Petite’s nearly 1-hour long series of passes back and forth between the two towers. Both include moments of rapidly rising tension and both have a clear line of resolution with a successfully concluded walk. The semantic content of the two clips is notably similar. During most of the action in the two sequences, the visual language concentrates on the protagonist on the wire. Slow pans over the wire, close-ups on Petite’s feet, or medium close-ups on his upper body provide information about his physical and mental state (pride and fear, most typically) with eventual cuts to his accomplices and other observers. The balance between dark- and bright-toned images divides the pre- and postcoup events from the actual wire-walking, as do day and night. Whereas the first clip opens with events taking place at an indoor space at dawn and continues with the performance in daylight, the second one presents the act first and then finishes indoors in the evening. There are some narrative cues that suggest the sequential order of the two clips in the movie when watched in its entirety; still, each sequence presents a stand-alone storyline without clearly referencing the other. This made the order of the two clips reversible and suitable for measuring participants for the same kinds of reactions while avoiding repetition and biases due to sequential effect.

### Participants

Thirty-eight volunteers, aged 24–37 (*M* = 28.6, *SD* = 3.52), were recruited for the experiment through academic and student organizations at Aalto University and word of mouth. All the participants were required to have normal or corrected-to-normal visual and hearing abilities and to possess sufficient skills in English. In addition, those who reported a lack of experience with smartphones (i.e., no access to or less than 2 months of experience; no consumption of audiovisual content on any portable smart devices) or other biasing factors were not considered for the experiment. Participants provided written informed consent according to the research protocol approved by the Aalto University Research Ethics Committee and received compensation for their time.

### Apparatus and Setup

In the projector condition, a stationary screen was used with a fixed viewing distance: Participants were seated in a shielded and dimmed (but not completely dark) experiment room at a fixed distance of 180 cm from a 47.3-inch (120 cm by its diagonal) canvas. The movie clip was projected on the canvas at a 32.4-degree horizontal^2^ and 18.55-degree vertical angle, and eye level was set to approximately the middle of the screening area. The visual angle was set in a way so as to exceed the range of angles for the mobile condition, even if participants hold the smartphone close to their eyes. For sound presentation, a pair of Sennheiser 400 headphones was provided with no noise-cancelling function.

Modelling the parameters of a typical smartphone viewing setup, the mobile condition was designed to recreate ordinary mobile viewing settings. For this reason, participants held the smartphone in their hands in a way that they found comfortable. They were permitted to adjust this position as well as the viewing distance between arm length and their eyes (approximately 60 cm). Therefore, the viewing distance varied between approximately 30–60 cm, which resulted in a horizontal angle of 11.52–22.8 degrees and a vertical angle of 6.49–12.93 degrees. For this setup, a 5.5-inch (13.9 cm diagonal) OnePlus 2 smartphone was provided, running Android 6.0 with 1,080 × 1,920 pixels of screen resolution. The phone was set to airplane mode so that the device could not generate any unforeseen distraction. The movie sequences were played on MX Player Pro video player application. The volume of the audio was synchronized to match that of the projector condition, and the same headphones were used.

In the interrupted conditions, additional audio and visual effects were played at determined points in time in correlation with the movie sequence. The time marks for distractors were assigned to specific narrative elements with meaningful or high emotional content and were the same for each and every participant. The specific distraction effects were chosen to model any unenclosed viewing space and, although they went off unannounced, created no more physical discomfort to participants than any stimuli in any natural environment. Three sonic and two visual distractors were used with varying source locations, durations, complexities, and ecological connection to the movie or the physical space. The specific distractors included a city sound with traffic noise (14 seconds), a ringing telephone (11 seconds), a written literary text (28 seconds), and bird chirping sound accompanied by an animated two-dimensional rectangle (9 seconds).

Separate speakers and a screen were used to play the distractors. The first distractor (traffic noise) was played from a parametric (directional) speaker, which threw sound in a relatively small, concentrated area, towards where the participant was seated. Sound arrived from behind and to the left of the participant. The second and the final sonic distractors (ringing phone and chirping birds) were presented from another, regular speaker in front and to the right of the participant. A 13-inch external screen was used for the visual distractors (literary text and animated rectangle), which was placed in front of the participant on the left. The luminance of the screen was set bright enough to be sensed, even if it was not in the viewer’s visual range (approx. 300 cd/m^2^). The external screen covered a 16.75-degree horizontal and 9.53-degree vertical angle.

For the projector conditions, the primary (movie clip) and secondary (distractor) stimuli were presented from the same computer using Presentation stimulus-presentation software by Neurobehavioral Systems. For the mobile conditions, Presentation scenarios contained all the respective stimuli, but the movie clip was controlled by the participant holding the smartphone. In both cases, the scenarios were coded to synchronize the clip and the distractors so that distractors would go off at the exact same moment of the clip for each participant. For this reason, time triggers were used that marked the start and end of the movie clip as well as the time of distractors. These triggers were sent to the respective measuring software for eye tracking (SMI BeGaze) and EDA (MegaWin by Mega Electronics) to avoid latencies between the different types of data.

### Procedure

After being recruited, each volunteer gained access to a short online survey that recorded demographic data, user habits, and experience with smart devices and mobile video player applications. Eligible volunteers were randomly assigned to two of the four conditions to watch the assigned clips. Each participant was tested individually.

Following an oral briefing, participants were seated in the experiment room, and the measuring tools (eye-tracking glasses and EDA skin sensors) were applied. The eye-tracking appliance was initially calibrated with one (central) calibration point, and if the participant’s gaze points showed at least approximately 0.5 degrees of deviation from the control fixation point, an additional, three-point calibration was used. The clips were presented with 5 seconds of black screen at the beginning to prepare the participant. Another black screen appeared for 5 seconds after the movie clip to signal the end of the trial.

In the projector conditions, participants received no specific instructions, other than to pay close attention to the movie sequence. In the mobile conditions, they received the same instruction but were also given the opportunity to exploit the functions of the video player application, interact with the device, and adjust the presentation of the sequence if and whenever they wished or felt the need to do so. The possibilities for adjustments are accounted for as part of the mobile conditions’ framework. No further analysis is conducted on interactions with the device.

After watching each assigned movie clip, participants were asked to complete a questionnaire that measured emotional engagement and presence and to answer questions regarding their comprehension of the movie content. In total, the experiment took no more than 45 minutes per participant including briefing, the two trials, and filling out a questionnaire after each trial.

### Measurements and Data Processing

Seven trials from the analysis of eye movements (9%) and 14 trials from the analysis of EDA (18.5%) were excluded due to technical errors or insufficient data. The resulting missing data can be classified as “missing completely at random” as data loss occurred irrespective of experiment conditions and were related to randomly occurring technical failures (Rubin, 1976; van Buuren, 2018). For the analysis of self-report of engagement and narrative comprehension, all trials were used for the final analysis.

#### Attention

Measuring the physiological factors of attention using eye tracking, the following indices were considered: the amount of time participants’ gazes were on the respective screen, the amplitude and frequency of saccades, and the dispersion of fixation points. Some of these indices overlap in measuring visual attention and information search during movie watching. They, however, help quantifying the various effects of screen type and environmental distractions by providing information on how distractions and a small handheld screen would impact attention oscillation between the movie and the physical space, and how that affects task complexity. Analysing the range of indices offers an opportunity to compare their effectiveness for the case of smartphone spectatorship.

For monitoring oculomotor behaviour, a pair of head-mounted SMI 1 mobile eye-tracking glasses was used with a sampling rate of 30 Hz. The mobile eye tracker enabled participants to move freely and interact naturally with the smartphone while registering both on-screen and off-screen gazes. Participants’ behaviour and device use were recorded by the high-definition video recorder built into the eye tracker; additionally, activity on the smartphone screen was monitored through screen capture, using AZ Screen Recorder Android application.

To measure the likelihood of participants’ gaze leaves the screen, a single dynamic area of interest (AOI) was defined that covered the respective screen on the eye tracker’s recording, independently of head movements and changes in the visual field. The respective AOI for each trial was set manually in SMI BeGaze (the eye tracker’s data recording and analysis software) to follow changes in position. This required adjusting the positions (*x* and *y* coordinates) of the four corners of the rectangular AOI manually frame by frame to align with the position of the screen as there was a lack of linear or automatically predictable movements. Being present throughout the entire trial, the AOI enabled distinguishing among all gaze activities that fell on or outside of the screen.

#### Arousal

EDA measures changes in skin conductance, which are closely related to emotional arousal, immersion, and attention. EDA was measured with sensors attached to participants’ fingers, which were connected to a digitizer (MegaWin ME6000 Biomonitor) with a sampling frequency of 1000 Hz. Data were recorded in microsiemens (µS). The sensors were placed on two fingers of a participant’s nondominant hand, on an area with a high density of sweat glands that would not interfere with carrying out the experiment tasks.

EDA produces a high variability of baseline levels in skin conductance in and between individuals depending on physiological responsiveness and skin type. For this reason, relative differences (percentages) were calculated between a baseline value and individual data points throughout the trials (Boucsein, 2012; Braithwaite et al., 2015). The baseline was the average EDA value of a 5-second window (5,000 data points) immediately preceding the start of the trial, when participants were not engaged in any tasks and were looking at the black screen. The percentages of changes from the baseline were used for the statistical analysis.

#### Subjective Ratings of Engagement

Participants evaluated their subjective impressions of their viewing experience on a 10-point Likert type scale with values ranging from *true* to *not at all true*. This questionnaire aimed to reveal engagement with the narration and general experience through the following indicators: presence in the diegetic space, empathy towards the characters, and levels of feeling scared, moved, and nauseated.

Major constructs from previous research were combined into one question (statement) for each item (Gross & Levenson, 1995; Qin et al., 2009; Witmer & Singer, 1998). The items to measure immersion (presence), emotional devotion (empathy), and the mental and bodily manifestations thereof (fear, being moved, nausea) were developed for the particular case of smartphone spectatorship. Wording followed first-person statements regarding the entire viewing experience with phrases as “I felt like I was present at the performance,” “I empathized with the actions of one or more character(s),” or “I felt scared/moved/nauseated.” To evaluate subjective ratings, each item of the questionnaire was analysed as an individual variable. An additional variable was calculated to determine individual averages of these ratings. A reliability test revealed an adequate consistency between the items with a Cronbach’s alpha of 0.772.

#### Narrative Comprehension

To assess narrative comprehension, participants responded to seven statements for each movie sequence relating to semantically meaningful narrative information and details that were obscured by the distractors in the interrupted conditions. The statements included, for example, “As a punishment, the wire walker had to perform another wire-walking act” or “There were two men who took pictures from the tower.” The details that present the correct answer to the statements were presented in the given sequence only once.

The possible answers to each question were *true*, *false*, and *I don’t know*. Answers were classified and analysed as “correct,” “incorrect,” and “I don’t know.” An “I don’t know” answer signalled a gap in accessing the relevant information, while an incorrect answer signalled an error. In other words, when a participant chose the “I don’t know option,” they were aware of the gap of knowledge, whereas choosing an incorrect answer meant that they comprehended/recalled information incorrectly. Thus, to compare participants’ performance, an overall score was calculated for each trial: Each correct answer equalled one point, and “I don’t know” answers equalled zero point; for incorrect answers, one point was deducted. The overall score was normalized (where necessary, missing values were replaced with the participant’s mean score).

## Results

To analyse the data processed with the aforementioned methods, a generalized linear mixed model analysis was performed, which included screen type and the presence or absence of distractors as independent variables. This analysis served the same purpose as a repeated measures analysis of variance, but it allowed for including participant as a random effect to control for the incomplete design (each participant being measured in two of the four conditions) while maximizing statistical power. The analysis was run for each dependent variable of attention, arousal, subjective ratings of engagement, and narrative comprehension. This determined the following effects of viewing conditions (see Table 1).

**Table 1.** Generalized Linear Mixed Model, Mean Values, Standard Deviations, Interactions, and Main Effects of Viewing Conditions on the Attention, Arousal, Engagement, and Narrative Comprehension.

Measure	Mean (standard deviation)				Unit
	Interrupted mobile	Uninterrupted mobile	Interrupted projector	Uninterrupted projector	
Dwell time	393,024.461 (54,612.819)	391,832.964 (59,868.034)	411,271.248 (58,410.398)	403,192.608 (66,904.644)	Millisecond
Average saccadic amplitude	4.828 (7.311)	2.683 (0.955)	7.789 (16.402)	5.391 (7.002)	Degree
Minimum saccadic amplitude**	0.337 (0.014)	0.337 (0.016)	0.34 (0.023)	0.35 (0.025)	Degree
Maximum saccadic amplitude	583.325 (1660.429)	34.774 (80.234)	1309.394 (5535.282)	2050.674 (8197.428)	Degree
Saccadic frequency*	1.889 (0.372)	2.136 (0.422)	2.124 (0.489)	1.889 (0.294)	Count/second
Gaze dispersion**	121.197 (43.723)	102.195 (42.93)	191.754 (95.054)	205.986 (143.115)	Standard deviation of coordinates
Electrodermal activity**	–0.011 (0.215)	–0.004 (0.221)	0.145 (0.319)	0.173 (0.336)	Percentage of baseline
Experience rating—presence***	4.389 (1.852)	6 (2.362)	4.6 (2.563)	4.833 (2.771)	–
Experience rating—empathy***	4.111 (1.906)	4.95 (1.905)	3.65 (2.301)	4.333 (2.142)	–
Experience rating—feeling scared	5.556 (2.64)	5.7 (3.08)	5.8 (3.002)	6.056 (2.999)	–
Experience rating—feeling moved	5.278 (2.191)	5.7 (2.515)	4.7 (2.155)	4.889 (2.324)	–
Experience rating—feeling nauseated	7.556 (3.382)	7.2 (2.783)	6.8 (3.071)	7.611 (3.5)	–
Experience rating—average	5.378 (1.585)	5.91 (1.807)	5.11 (1.856)	5.544 (1.477)	–
Narrative comprehension*	1.111 (2.42)	3.255 (2.053)	2.4 (2.371)	2.153 (1.935)	–

*Note*. Eye tracking measures were performed using a sample size *n* = 69, electrodermal activity measures *n* = 62, and experience ratings and narrative comprehension *n* = 76.

* Significant interaction; **Significant main effect of screen; ***Significant main effect of distraction.

### Attention

The first research question asked about the effects of viewing conditions on gaze behaviour. We hypothesized viewers’ gazes to travel longer and leave the screen for a longer proportion of the trial time in the presence of distraction and when watching the movie clip on the smartphone screen.

#### Dwell Time on Screen

We expected that interrupted mobile viewing likely distracts attention from the movie, which decreases the time spent on the screen AOI. However, running the model on dwell-time data, results showed no significant interaction between the effects of screen type and the presence or absence of distraction, *F*(1, 65) = 0.004, *p* = .949. Screen type, *F*(1, 65) = 1.958, *p* = .166, and distractions, *F*(1, 65) = 1.25, *p* = .268, had no significant main effects either. Results of this test indicated that dwell time and, thus, attention to the respective screen was not affected by screen type or the presence of distractors. This result was confirmed by a complementing analysis of the frequency of off-screen fixations that measured the proportion of gaze events that fall outside the screen: The frequency of off-screen fixations was similarly unaffected by screen type or the presence of distractors.

#### Saccadic Amplitude

According to our hypothesis that viewers’ gaze would likely leave the screen in the interrupted conditions, we expected a general increase in saccadic amplitude compared with uninterrupted viewing. Similarly based on the longer gaze trajectory, we predicted higher average saccadic amplitude for the projector condition than the mobile condition. In addition to an average value of saccadic amplitude, we also compared maximum and minimum values. Maximum values are constrained to the area that contain relevant information (a larger screen covers a larger area and distractions may further extend the area of visual search). Minimum values can be more sensitive for determining the effects of viewing conditions on visual search.

Average and maximum values showed no significant interaction between the effects of screen type and the presence or absence of distraction, *F*(1, 64) = 0.003, *p* = .953 and *F*(1, 65) = 0, *p* = .998. On average and maximum values, neither the screen type, *F*(1, 64) = 1.658, *p* = .203 and *F*(1, 65) = 0, *p* = .996, nor the distraction, *F*(1, 64) = 1.06, *p* = .307 and *F*(1, 65) = 0, *p* = 1, had significant main effects.

Although average and maximum values were independent of viewing conditions, comparing the minimum values of saccadic amplitude partly confirmed our hypotheses: A significant main effect was observed between screen types, *F*(1, 65) = 4.137, *p* = .046. The minimum values of saccadic amplitude were significantly higher for the projector conditions than for mobile conditions. No significant interaction, *F*(1, 65) = 0.872, *p* = .354, and no significant main effect of distraction, *F*(1, 65) = 1.696, *p* = .197, were observed for minimum saccadic amplitude.

#### Saccadic Frequency

Measuring the number of saccades per second, the results revealed that variables interacted in their effect on saccadic frequency, *F*(1, 65) = 4.306, *p* = .042. A simple main effect analysis determined that saccadic frequency was significantly higher for the mobile screen during uninterrupted viewings, meaning that participants performed more saccades when watching the clip on the mobile screen than on the projector screen in the absence of distractions, *t*(65) = 2.162, *p* = .034.

#### Gaze Dispersion

According to our hypothesis, fixation points^3^ were expected to be more concentrated in the central area of the image on the smartphone than they are on the large screen. To quantify and compare the variation (dispersion) of fixation coordinates, the standard deviation of all fixation coordinates was calculated for each trial. Here, a lower standard deviation value reveals that these points are distributed in a smaller area around the central point. The dispersion of fixation points decreases with smaller screens, so mobile conditions were expected to produce lower fixation dispersion.

As anticipated, screen type had a main effect on gaze dispersion, *F*(1, 64) = 26.229, *p* < .001. Fixations were spread on a significantly larger area when watching the projector screen than when watching the mobile screen. No significant interaction, *F*(1, 64) = 0.502, *p* = .481, or main effect of distraction, *F*(1, 64) = 0.024, *p* = .877, was observed.

### Arousal

Having explored the effects of attention and gaze behaviour, next we looked at arousal. EDA measurements provided an overall EDA score, the mean value per trial relative to the baseline value. A comparison of the corrected overall scores across conditions was anticipated to determine changes in arousal that originated from engagement with the movie clip and the diegetic events. This suggests that EDA values would be higher in the projector conditions and during uninterrupted viewing.

EDA values showed a main effect of screen, *F*(1, 58) = 5.78, *p* = .019, where the average EDA level was significantly higher for the projector conditions than mobile conditions. This result indicates that participants were more aroused during projector watching. No significant interaction, *F*(1, 58) = 0.014, *p* = .906, or main effect of distraction, *F*(1, 58) = 0.071, *p* = .791, was observed for this variable.

### Engagement

Following the third research question, we tested participants’ self-reported engagement. The individual average scores as well as all the separate items of the self-report questionnaire (sensation of presence, empathy, feeling scared, moved, and nauseated) were expected to have lower values for small-screen and interrupted trials.

In terms of the average value, results showed no significant main effect of distraction, *F*(1, 72) = 3.611, *p* = .061, or screen, *F*(1, 72) = 1.55, *p* = .217, or significant interaction, *F*(1, 72) = 0.01, *p* = .922. To further explore this finding, we ran the analysis on the individual items.

In the case of presence and empathy ratings, significant main effects of distraction were observed: Ratings for both items were significantly higher in the uninterrupted conditions than in the interrupted conditions, *F*(1, 72) = 4.644, *p* = .034 and *F*(1, 72) = 6.645, *p* = .012. However, results showed no significant interactions between the effects of screen type and distraction on presence and empathy, *F*(1, 72) = 1.102, *p* = .297 and *F*(1, 72) = 0.017, *p* = .898, and screen had no significant main effect on these items, *F*(1, 72) = 1.247, *p* = .268 and *F*(1, 72) = 3.331, *p* = .072.

Subjective ratings of feeling scared, *F*(1, 72) = 0.004, *p* = .948, being moved, *F*(1, 72) = 0.032, *p* = .859, or experiencing nausea, *F*(1, 72) = 0.375, *p* = .542, showed no significant interaction between screen type and the presence or absence of distraction. Neither the screen type, *F*(1, 72) = 0.477, *p* = .492; *F*(1, 72) = 3.731, *p* = .057; *F*(1, 72) = 0.18, *p* = .673, nor the distraction, *F*(1, 72) = 0.212, *p* = .647; *F*(1, 72) = 0.722, *p* = .398; *F*(1, 72) = 0.314, *p* = .577, had main effects on these variables.

### Narrative Comprehension

Mobile screen and interrupted viewing were hypothesized to decrease engagement with the movie and, consequently, poorer recollection of narrative details. For testing narrative comprehension, the overall scores indicating individual performance were compared. Narrative comprehension scores showed a significant interaction between screen type and distraction, *F*(1, 72) = 4.811, *p* = .032. A post hoc exploration of this interaction revealed a simple main effect of distractors, *t*(72) = 2.945, *p* = .004: Participants scored significantly higher in uninterrupted condition than interrupted condition when watching the movie clip on the smartphone.

## Discussion

In the present study, we tested the effects of screen type and environmental distractions on attention, arousal, engagement, and narrative comprehension during movie watching. The results revealed the impact of viewing conditions on eye movements (minimum saccadic amplitude and saccadic frequency), gaze dispersion, EDA, self-reports of engagement (feeling of presence and empathy), as well as comprehension.

Measuring the distance the eye travels between two fixation points, saccadic amplitude is of particular interest in assessing skewness in participants exploring the screen and off-screen areas. Viewers’ minimum saccadic amplitude was higher when watching a movie clip on the projector screen than on the mobile device meaning that their gaze travelled longer when viewing the projector. Correspondingly, the dispersion of gaze increased in the case of the larger screen: As expected, fixation points covered a larger proportion of the surface of the projector screen than the mobile screen. This even seems to comply with the fact that, although larger displays produce a larger retinal image, a greater proportion of the image stays outside the fovea, the area that provides sharp vision. For saccadic frequency, an opposing tendency was observed: Viewers moved their eyes more in the mobile condition but only in the absence of distractions. We explain this by the fact that viewers’ gazes travel shorter distances on a small screen: Screen content can be explored while making smaller changes to gaze position, which can increase the frequency of saccades.

Besides screen size, previous research has shown that saccadic behaviour correlates with the difficulty of task (May et al., 1990). More specifically, a complex task (for instance, difficulty focusing on a small handheld screen’s content while interrupted by distractors) likely decreases the amount of eye movements. This raises the question of how cognitively demanding smartphone viewing is or how complex a mental activity it involves. However, in contrary to our expectations, interrupted mobile viewing did not affect saccadic behaviour in a way that would suggest it being more demanding or complex than the more conventional fixed-screen viewing that encompasses a larger proportion of the history of moving-image media.

Neither the screen type nor the presence or absence of distractors showed an effect on the proportion of time participants’ gaze spent on or outside of the screen. This signals that viewers were similarly unlikely to transfer their visual attention to the surrounding space from a small portable screen as from a large stationary screen, even in the presence of environmental distractions. The lack of significant differences between viewing conditions in the tendency of off-screen gaze may suggest that viewers are able to accustom to viewing small handheld screens in unenclosed environments and engage in movie experiences comparable to large-screen viewing. A possible reason would lie in the affective quality of feature films; the idea that the complexity of the movie stimulus and the high perceptual load it entails would limit the perceptual resources to be directed to off-screen stimuli (Bailenson & Yee, 2006; de Kort & IJsselsteijn, 2008; Lavie, 1995). Other possible factors can be related to smartphone usage and the properties of the device. Active smartphone users (all participants had over 2 months of experience with consuming audiovisual content on their smartphones) might accustom to screens and viewing environments easily, which fosters a focused movie experience. Moreover, handheld control over the screen and its position might have led to an optimal and focused viewing, where visual attention concentrated on the screen can successfully mask external distractors. However, both the role of frequent smartphone usage and handheld screen control require further investigation in future research.

Immersion and narrative engagement may be measured by the level of arousal in response to narrative events (Boucsein, 2012; Potter & Bolls, 2012). EDA results showed that screen type has an impact on arousal. Arousal was expected to be lower in the mobile conditions than in the projector conditions, and the results corresponded with this expectation. This suggests that a larger screen increases viewers’ involvement with a movie and that this involvement leads to a higher level of responsiveness to narrative events.

The EDA results stand in accordance to the increased engagement with moving images on bigger screens found by Freeman et al. (2000), IJsselsteijn et al. (2001), Troscianko et al. (2012), and others. Yet, screen size did not affect the subjective indices of engagement in the present study the same way it did in previous research. This result might signal that handheld smartphone screens impact viewing experiences differently than stationary screens of various sizes. In addition, although we did not account for the changes of visual angle in the design of this experiment, it is possible that these factors affected our results in that mobile participants’ subjective sensation of engagement increased with the control of the screen to the level of projector participants’.

Distractors, however, had effects on the subjective assessments of presence and empathy: Participants in the interrupted conditions rated their sensation of presence and empathy lower than those in the uninterrupted conditions. The impact of distractors shows that environmental stimuli can make it more difficult to engage with a fictional world and its characters. A similar effect of distractors was observed for the case of narrative comprehension: Participants scored lower in the comprehension test in the presence of distractors when watching the movie clip on the smartphone.

### Limitations

We demonstrated differences between smartphone and stationary screen viewing in unenclosed environments in some variables and not in others. Here, it is worth reflecting on the fact that viewers wore eye-tracking glasses that may have impacted on the “natural viewing.” For example, they may have blocked the complete view of the visual distractors appearing on an external screen at an approximately 45-degree angle upwards when the movie was being played on the smartphone. Nevertheless, the use of eye tracking in the current study makes an important contribution in combination with our other measures.

In this experiment, we used physiological measurements, performance tests, and self-reports. This combination of objective and subjective measurements is able to capture viewer behaviour even when subjective responses are biased by social and cultural expectations or mental abilities, such as memory and language skills. Still, we cannot rule out that some of the results are content specific and cannot be generalized beyond the movie sequences used here. Previous research provides evidence on the correlation between the emotions and attention (Fredrickson & Branigan, 2005; Rowe et al., 2007). This tendency was demonstrated for the case of movie watching: Finucane’s (2011) study shows that watching fearful movie sequences narrows attention. Bezdek and Gerrig (2017; see also Bezdek et al., 2015) came to corresponding conclusions finding that suspense (the potential of negative future events) in films increases narrative transportation and, therefore, the accuracy of recollection and decreases attention to external stimuli or secondary tasks. As the film clips used in the present study evoke suspense and present scenes with intense emotional tension (i.e., fear for the main protagonist’s unsuccessful wire-walking act and death), the narrative itself and its emotional content may have affected our results in terms of attention to the movie, lack of attention to external stimuli, arousal, and engagement. To determine the specific effects of film genre, emotional content, and certain semantic information (faces, bodies, urban environments, or even the effects of acrophobia) on handheld and stationary screen viewing, additional tests are required.

Potential future studies include a fine-grained analysis of the way adjustments of the mobile screen’s position and other interactions would affect viewer responses. In addition, our focus was on individual viewing strategies, but another potentially significant factor of unenclosed viewing environments is the proximity and social presence of others. Testing viewer behaviour in the presence of others is also subject to further research.

## Conclusion

In conclusion, our results show that screen type has effects on the physiological indicators of attention and arousal, and distractions have effects on the sensation of engagement and narrative comprehension. These findings imply that viewers can comprehend narrative information and engage with a fictional narrative better when there are no distractions but also that watching large stationary screens in designated viewing environments increases engagement with the movie. From another angle, smartphone viewers are more likely to be affected by external distractions, which effect was foremost observable in terms of subjective ratings and narrative comprehension. These results confirm the importance of regarding smartphones as distinct media tools that encompass specific practices and clear-cut impact on viewing experiences.

## Notes

1 On reactions being biased due to knowledge of a sequence, see Noton and Stark (1971a, 1971b).

2 For the sake of comparison, a viewer seated on the prime seat of a movie theater (the area sold or occupied first, approximately at the back two thirds of an auditorium) sees the screen with an average horizontal angle of 45 degrees (Allen, 1999).

3 Gaze events with a minimum duration of 80 milliseconds were treated as fixations.
